# Commentary: Evaluating the efficacy and safety of tebentafusp in the treatment of metastatic uveal melanoma: a 2025 update systematic review and meta-analysis

**DOI:** 10.3389/fonc.2026.1767211

**Published:** 2026-01-30

**Authors:** Jean Henri Maselli-Schoueri, Samuel David Saibil

**Affiliations:** Princess Margaret Cancer Centre, Division of Medical Oncology & Hematology, Department of Medicine, University of Toronto, Toronto, ON, Canada

**Keywords:** meta - analysis, methodology, systematic review, tebentafusp, uveal melanoma

## Introduction

We read with great interest the article by Wang et al., which further expands our understanding of the efficacy and safety profile of tebentafusp for the treatment of metastatic uveal melanoma (1). Given the rarity of the disease, which has an annual incidence of approximately 5 cases per million inhabitants in Western populations, meta-analyses remain essential to contextualize clinical outcomes across studies (2). However, obtaining robust conclusions warrants careful methodological rigor in study selection, classification, and statistical modeling.

## Study classification, potential duplication and inappropriate modeling

First, the present paper identifies three randomized controlled trials (RCTs) in Table 1; however, upon further review, this classification does not reflect the actual study designs. The pivotal IMCgp100–202 trial represents a single RCT, not two different studies (3, 4). Furthermore, Piulats et al. performed a propensity-score-weighted comparison of tebentafusp versus immune checkpoint inhibitors, which, despite attempts to adjust for confounding, remains observational (5). Likewise, Maurer et al. reported a retrospective cohort, which is also non-randomized (6). Therefore, the present article effectively included only one RCT, and it appears from Figure 2 that patient data from the same trial were counted twice (baseline vs 3-year update), which could influence the pooled estimates and artificially reduce interstudy heterogeneity.

Second, upon further review of the results section and Figure 2A, an inconsistency becomes apparent. The text mentions a pooled complete response rate of 0.01 (95% CI −0.00 to 0.01). If this is intended to represent pooled analysis, the negative lower limit indicates that the analysis was performed on an inappropriate statistical scale. Proportions by definition are bounded between 0 and 1 – therefore, confidence intervals must remain within this range. Applying a variance-stabilizing transformation such as logit, double-arcsine or other techniques would have prevented this artifact and yielded interpretable intervals within the expected and mathematically appropriate 0–1 range (7, 8). Alternatively, if there is a mismatch between the text and the figure, and the latter represents only a risk difference, then the results might be numerically negative, but they would be clinically uninterpretable in the context of a single-arm meta-analysis.

Third, the analysis also reports “pooled median PFS and OS,” which might be methodologically unsound in this context. Pooling medians across studies ignores the shape and censoring of survival curves. This practice may distort the true variability in outcomes, particularly when follow-up durations differ: medians describe moments; hazard ratios describe processes. Thus, the appropriate metric would be the hazard ratio or reconstructed individual-level time-to-event data (9, 10). Furthermore, it is worth noting that subgroup comparisons between “first-line” and “pretreated” cohorts are not consistently defined and likely affected by confounding factors such as hepatic tumor burden and dosing schemes. Taken together, the aforementioned issues highlight areas in which methodological clarification could strengthen the interpretation of results presented in the paper. However, at the same time, we recognize the challenges inherent in synthesizing evidence in such a rare disease, and this does not detract from the overall contribution of Wang et al. In this context, our group independently conducted a systematic review and meta-analysis with results that, despite mathematically different, point toward a similar direction, indicating that for patients with metastatic uveal melanoma that are known to be HLA-A*02:01-positive, tebentafusp was associated with both improved survival outcomes and acceptable toxicity (11).

## Conclusion

Wang et al. faced a difficult challenge: to perform a meta-analysis on a very rare disease. The authors rightly call for the need for broader datasets and highlight the emerging role of ctDNA as a biomarker. However, a few methodological considerations, if clarified, would strengthen the interpretation of tebentafusp’s efficacy profile. In the end, science moves forward – sometimes haltingly – but only when it walks on firm methodological ground.
